# “She’ll Be Right, Mate”: A Mixed Methods Analysis of Skin Cancer Prevention Practices among Australian Farmers—An At-Risk Group

**DOI:** 10.3390/ijerph19052940

**Published:** 2022-03-03

**Authors:** Camilla Trenerry, Chloe Fletcher, Carlene Wilson, Kate Gunn

**Affiliations:** 1 School of Psychology, The University of Adelaide, Adelaide, SA 5000, Australia; camilla.trenerry@sa.gov.au; 2 Freemasons Centre for Male Health and Wellbeing, The University of Adelaide, Adelaide, SA 5000, Australia; 3 Flinders Health and Medical Research Institute, College of Medicine and Public Health, Flinders University, Bedford Park, SA 5042, Australia; carlene.wilson@flinders.edu.au; 4 Department of Rural Health, Allied Health and Human Performance, University of South Australia, Adelaide, SA 5000, Australia; chloe.fletcher@unisa.edu.au; 5 Olivia Newton John Cancer Wellness Research Centre, Austin Health, Heidelberg, VIC 3084, Australia; 6 School of Psychology and Public Health, La Trobe University, Bundoora, VIC 3083, Australia

**Keywords:** farm, agriculture, skin cancer, rural health, occupational medicine, cancer prevention

## Abstract

This study examined Australian farmers’ engagement with skin cancer prevention behaviours and explored what made it hard for them to be ‘SunSmart’ (barriers), and what could be done to make prevention easier (facilitators). In total, 498 farmers (83.1% male, 22–89 years, 50.8% grain, sheep, or cattle farmers) participated. The least frequently performed SunSmart behaviours (reported as never practiced during summer) were using SPF 30+ sunscreen (16.6%), wearing protective sunglasses (10.5%), and wearing protective clothing (8.6%). Greater engagement (i.e., higher scores on scale from Never to Always) with SunSmart behaviours was explained by gender (female), educational attainment (trade or technical college certificate vs. high school), personal skin cancer history, and skin sun sensitivity. Barriers reported by farmers related to personal preferences (e.g., short-sleeved rather than long-sleeved clothing), comfort, and perceived impracticality of sun protection. Farmers’ solutions included making protective clothing and sunscreen more appropriate for farm work (e.g., by making clothing more breathable). A personal health scare was the most reported motivation for skin cancer prevention. Findings highlight the need for increased access to sun-protective clothing and sunscreen that is suitable for wearing when working on farms, complemented by culturally appropriate health education messaging, to encourage more farmers to perform SunSmart behaviours.

## 1. Introduction

Solar ultraviolet radiation (UVR) levels are estimated to be 3 to 5 times higher in Australia than other parts of the world [1,2]. Exposure to solar UVR is thought to play a causative role in 65% of melanoma and 90% of non-melanoma skin cancers [3,4]. Moreover, the risk of developing skin cancer increases with the level of exposure to solar UVR and, in particular, with repeated exposure over time [5]. Australia, being located near the equator, also tends to be warm and conducive to outdoor recreational and occupational activities. Because of this, people living in Australia spend more time outdoors during leisure time, and typically wear less clothing, increasing their exposure to solar UVR. Consequently, Australia has one of the highest rates of skin cancers in the world [6,7]; 80% of all newly diagnosed cancers are melanoma or non-melanoma skin cancers (referred to as NMSC, which includes keratinocyte cancers such as basal cell carcinoma and squamous cell carcinoma) [8]. It is estimated that two in three Australians will be diagnosed with skin cancer by 70 years of age [9,10].

Farmers, who work mainly outdoors, experience chronic occupational solar UVR exposure [11]. The level of exposure they experience is estimated to be six to eight times greater than that of indoor workers [12]. It is therefore unsurprising that the risk of skin and lip cancers is significantly higher among farmers and agricultural workers compared to the general population [13,14]. The risk is likely to be even higher for farmers who are fairer skinned and more sensitive to the sun, in comparison with those who are darker skinned and less sun-sensitive [5]. Australian farmers are 60% more likely than the general population to die from melanoma and other skin cancers [15]. For older farmers, this disparity is even greater; those who are 65 years or older die of all types of skin cancer at more than double the rate of other Australians [16]. The reasons for this may be structural (e.g., availability of suitable skin checking services, or delays in accessing services and therefore diagnosis due to waiting times), knowledge-related (e.g., inadequate health literacy on the risks of skin cancer for outdoor workers), or attitudinal (e.g., stoicism and other socio-cultural factors).

Fortunately, there are simple ways to reduce exposure to solar UVR. Cancer Council Australia launched their Slip! Slop! Slap! campaign in 1981 [17], successfully increasing awareness, changing attitudes, and encouraging Australians to practice sun-protective behaviours (e.g., ‘slipping’ on a shirt, ‘slopping’ on sunscreen, and ‘slapping’ on a hat) [18,19]. The original slogan, which has become the core message of Cancer Council’s SunSmart program [20], has since been updated to include two additional sun-protective behaviours: ‘seeking’ shade when possible and ‘sliding’ on wraparound sunglasses to prevent sun damage to the eyes [17]. The SunSmart program has not only helped to modify individual behaviour, but also to shape legislation and public policy (e.g., by integrating skin protection into work health and safety regulations) and infrastructure (e.g., by introducing sun-protected environments in workplaces and public areas) [21]. 

Despite the general success of these campaigns, research examining sun-protective behaviours among farmers suggests that the messages have not adequately engaged farming populations [11,22,23,24]. Farmers are known to be reluctant to prioritise preventive health [25,26], and despite Smit-Kroner and Brumby’s [11] finding that sunscreen was the most studied, most reported, and most promoted component of the SunSmart program, a number of studies show that farmers rarely use it [22,23,24,27]. Farmers in Australia and internationally report a range of barriers to sun protection that are specific to their working conditions; discomfort wearing long-sleeved clothing in the heat, and dust and dirt sticking to sunscreen [22,27,28,29]. Barriers may be further compounded by underestimation of skin cancer risk [22,30,31]. More research is needed to gain a better understanding of Australian farmers’ engagement with sun-protective behaviours, including the specific factors that prevent, facilitate, and motivate them to make skin cancer prevention a priority. Currently, what is missing from the literature is clear insight, from the perspective of Australian farmers, into how barriers to engaging in sun-protective behaviours could be overcome. The aims of the present study are to (1) explore farmers’ engagement with skin cancer prevention behaviours, (2) examine how prevention behaviours vary by gender, age, education, farming type, personal and family history of skin cancer, and skin type, and (3) explore the barriers, facilitators, and motivators for engagement with skin cancer prevention, with a particular focus on farmers’ ideas about how barriers could be addressed. In doing so, this study will inform the development of strategies to help reduce the rate of skin cancer, in this at-risk population. 

## 2. Materials and Methods

### 2.1. Ethical Approval

Study approved by the University of Adelaide’s School of Psychology Human Research Ethics Committee (HREC-2015-47). 

### 2.2. Design

A cross-sectional, mixed methods design was used to assess farmers’ engagement with skin cancer prevention behaviours and explore the barriers, facilitators, and motivating influences on sun protection. Data were collected were collected as part of a student project, conducted within a fixed timeframe, and via paper-based surveys that included closed- and open-ended questions. Demographic information was also collected to describe the sample and facilitate identification of correlates of engagement with prevention behaviours.

### 2.3. Participants and Recruitment Strategy

Farmers were eligible to participate in the study if they were aged 18 years or over, understood and spoke English, and worked outdoors on a farm, or in a livestock or pastoral enterprise. Farmers were recruited through Livestock SA, a membership-based organisation representing the interests of beef cattle, sheep, and goat farmers in South Australia. Livestock SA members were emailed a notice one month prior, advising them of the intention to mail out paper-based surveys. Completed surveys were returned in reply-paid envelopes by 501 farmers. The response rate was 30.3%, which is pleasing given challenges with recruitment in this population. For example, Gunn, et al. [32] obtained a response rate of 14.7% for a study examining Australian farmers’ stress and coping during drought, and Bennett and Cattle [33] obtained a response rate of 20% for their study examining adoption of soil health practices. Three surveys that were completed together with a spouse were excluded from the analyses. Demographic characteristics of the sample were comparable with those of Australian farmers more broadly [34].

### 2.4. Measures

Ten items assessed farmers’ demographic characteristics, including predisposition to skin cancer (i.e., Fitzpatrick skin photo-type, personal history and family history of skin cancer), personal attributes (i.e., gender, age, marital status, education level achieved), regional location and farm type. The Fitzpatrick skin photo-type scale [35] was developed to assess an individual’s skin colour and their tendency to burn or tan when exposed to UVR from the sun. Classifications of skin photo-type were based on response to the following question: ‘which of the following would best describe your reaction to your first exposure to summer sun, without sunscreen, for half an hour at midday?’ Response options ranged between (1) always burn, unable to tan and (6) almost never burn, deeply pigmented.

Five items assessed farmers’ performance of the SunSmart skin cancer prevention behaviours, in line with Cancer Council Australia recommendations. These were: (1) wearing protective clothing (e.g., a long shirt and pants), (2) wearing SPF 30+ (or higher) sunscreen, (3) wearing a wide-brimmed hat, (4) seeking shade, and (5) wearing protective sunglasses. Items were originally developed by White, et al. [36] but were adapted to describe each sun-protective behaviour according to Azjen’s [37,38] TACT principle (e.g., the target behaviour, the action involved, the context in which it occurs, and the time frame). Given the timing of data collection (winter), respondents were prompted to answer questions in relation to their behaviour during the past summer. Respondents were asked: in general, how often did you perform the following behaviours to protect yourself from UVR when you went outside for more than 10 min? Items were rated on a 7-point Likert scale where 1 = never and 7 = always.

Three open-ended questions were included to explore barriers, facilitators, and motivating influences on skin cancer prevention behaviours. These were: (1) What made performing one or more of the skin cancer prevention behaviours outlined above difficult for you last summer? (2) How could the skin cancer prevention behaviours listed above be made easier to perform? (3) What would motivate you to make preventing skin cancer a priority?

### 2.5. Analysis

#### 2.5.1. Quantitative Data

Quantitative data were analysed using IBM SPSS Statistics (2019, standard version 26). Frequencies described prevalence of prevention behaviours. Shapiro–Wilk tests indicated data were not normally distributed, therefore non-parametric tests were used to examine differences in engagement with prevention behaviours across demographic variables. Mann–Whitney U tests were used to examine differences between groups based on gender (which was measured dichotomously), whereas Kruskal–Wallis H tests were used to examine differences between groups based on age, education, personal and family history of skin cancer, farm type, and Fitzpatrick skin photo-type category. 

#### 2.5.2. Qualitative Data

Responses to open-ended questions were analysed by CF and refined with input from KG, using NVivo 11 Plus [39] to help organise the data. Qualitative content analysis was employed to identify farmers’ reports of barriers, facilitators, and motivating influences on prevention behaviours. Content analysis was chosen based on the nature of the qualitative data (short responses to open-ended survey questions) and because it employs a descriptive approach to the coding of data and interpretation of quantitative counts of codes [40]. A bottom-up, inductive approach was used, rather than applying a prescriptive list of assumed codes, to ensure that codes accurately reflected farmers’ experiences. 

Responses to each of the open-ended questions were analysed separately and were initially coded descriptively as a barrier, facilitator, or motivating influence. Throughout the analysis, subsequent responses were compared to previously coded text and were either allocated to an existing code or assigned a new one. When all text had been coded, codes were examined for similarities and differences in content. Those with similar content were grouped into categories to describe the key barriers, facilitators, and motivating influences. Coding trees are presented in Supplementary Materials. Categories were reviewed by the research team and any disagreement or uncertainty in coding was resolved through discussion.

## 3. Results

### 3.1. Participant Characteristics

Of the 498 survey respondents analysed, 85.9% reported working on a grain, sheep or cattle farm, or sheep and/or cattle property (Table 1). Participants’ ages ranged from 22–89 years (M = 56.4, SD = 11.05) and 83.1% identified as male. Most (88.8%) reported being married or living with a partner. Participants had diverse educational backgrounds, with 29.9% completing a trade or TAFE certificate and 20.1% having a university degree. Only 5.4% of participants reported a personal history of melanoma, whereas 23.7% had a personal history of NMSC.

### 3.2. Frequency of Engagement with SunSmart Behaviours and Influences

As defined previously, skin cancer prevention behaviours were operationalised as wearing protective clothing (e.g., a long shirt and pants), wearing SPF 30+ (or higher) sunscreen, wearing a wide-brimmed hat, seeking shade, and wearing protective sunglasses. Farmers reported infrequent use of sunscreen in the preceding summer; 16.6% reported that they never wore sunscreen, and 51.4% reported that they did so less than half the time (i.e., participant rated items assessing performance of SunSmart behaviours as either 1, 2 or 3 on). Additionally, 8.6% of farmers reported that they *never* wore protective clothing during the past summer (and 26.2% reported doing so less than half the time). Wearing a hat or sunglasses were the most commonly performed behaviours. Frequencies for all skin cancer prevention behaviours are outlined in Table 2.

Analyses were undertaken to examine whether engagement with prevention behaviours differed according to farmers’ demographic characteristics, Fitzpatrick skin photo-type, and personal or family history. These results are presented in Table 3 and Table 4 and described below.

#### 3.2.1. Sunscreen

Sunscreen use was significantly higher among female farmers (mean rank = 313.38) than males (mean rank = 217.58), U = 6647.500, z = −5.180, *p* < 0.001. Sunscreen use also differed significantly across education groups, χ^2^(2) = 7.772, *p* = 0.021. Post-hoc pairwise comparisons with Bonferroni correction revealed that farmers who had completed TAFE or trade school (mean rank = 264.40) used sunscreen significantly more often than those who had only completed high school (mean rank = 226.02) (*p* = 0.022). Interestingly, mean ranks for sunscreen use were comparable between farmers who had completed high school (226.02) and those who had gone on to complete university or college (229.08). Sunscreen use did not vary by age (*p* > 0.05), suggesting that the aforementioned finding may be associated with attendance at TAFE or trade school. 

Farmer’s Fitzpatrick skin photo-type contributed to variation in sunscreen use (χ^2^(5) = 39.676, *p* < 0.000). Post-hoc comparisons confirmed that sunscreen use was significantly greater in farmers with skin type I (always burn; mean rank = 339.84) than those with skin types II (usually burn; mean rank = 259.65, *p* = 0.035), III (sometimes mild burn; mean rank = 244.68, *p* = 0.003), IV (rarely burn; mean rank = 195.52, *p* < 0.000), and V (almost never burn, tan deeply; mean rank = 170.42, *p* < 0.000). Farmers with skin types II (usually burn; mean rank = 259.65) and III (sometimes mild burn; mean rank = 244.68) also reported wearing sunscreen significantly more often than those with skin type IV (rarely burn; mean rank = 195.52) (p’s = 0.003 and 0.037, respectively).

Finally, personal history of skin cancer contributed to variation in sunscreen use (χ^2^(2) = 9.424, *p* = 0.009). Post-hoc pairwise comparisons revealed that farmers who had a history of NMSC (mean rank = 272.13) used sunscreen significantly more often than those who had a history of melanoma (mean rank = 213.61) or no history of skin cancer (mean rank = 229.55) (*p* = 0.044 and 0.004, respectively). Having a family rather than personal history of skin cancer was not associated with sunscreen use (*p* > 0.05).

#### 3.2.2. Protective Clothing

Wearing protective clothing varied between age groups (χ^2^(6) = 18.430, *p* = 0.005), with farmers aged 60–69 years (mean rank = 264.48) wearing protective clothing significantly more often than those aged 40–49 years (mean rank = 204.59) (*p* = 0.020).

Farmer’s use of protective clothing varied according to their Fitzpatrick skin photo-type (χ^2^(5) = 21.663, *p* = 001); post-hoc comparisons showed that farmers with skin type I (always burn; mean rank = 310.83) wore protective clothing significantly more often than those with skin types III (sometimes mild burn; mean rank = 227.44, *p* = 0.016) and IV (rarely burn; mean rank = 215.04, *p* = 0.005). Additionally, farmers with skin type II (usually burn) reported wearing protective clothing significantly more often than those with skin type IV (rarely burn) (mean rank = 272.56 vs. 215.04; *p* = 0.015). 

#### 3.2.3. Wide-Brimmed Hat

Kruskal–Wallis H test indicated that wearing a wide-brimmed hat varied between age groups (χ^2^(6) = 13.605, *p* = 0.034); however, when post-hoc pairwise comparisons (with Bonferroni correction) were conducted to identify which age groups these differences existed between, all adjusted p’s > 0.05. Personal history of skin cancer contributed to variation in wearing a wide-brimmed hat (χ^2^(2) = 18.869, *p* < 0.000); farmers who had a history of NMSC (mean rank = 288.46) reported wearing a wide-brimmed hat more often than those who had no history of skin cancer (mean rank = 226.15) (*p* < 0.000). 

#### 3.2.4. Protective Sunglasses

Wearing protective sunglasses varied significantly across age groups (χ^2^(6) = 21.046, *p* = 0.002), with farmers aged 40–49 (mean rank = 272.56) wearing protective sunglasses significantly more often than those aged 60–69 (mean rank = 211.14) and 70–79 (mean rank = 193.52) years (*p* = 0.009 and 0.021, respectively). 

#### 3.2.5. Shade

Seeking shade did not vary according to farmers’ demographic characteristics, personal or family history of skin cancer, or Fitzpatrick skin photo-type.

### 3.3. Qualitative Findings

**Figure 1:**
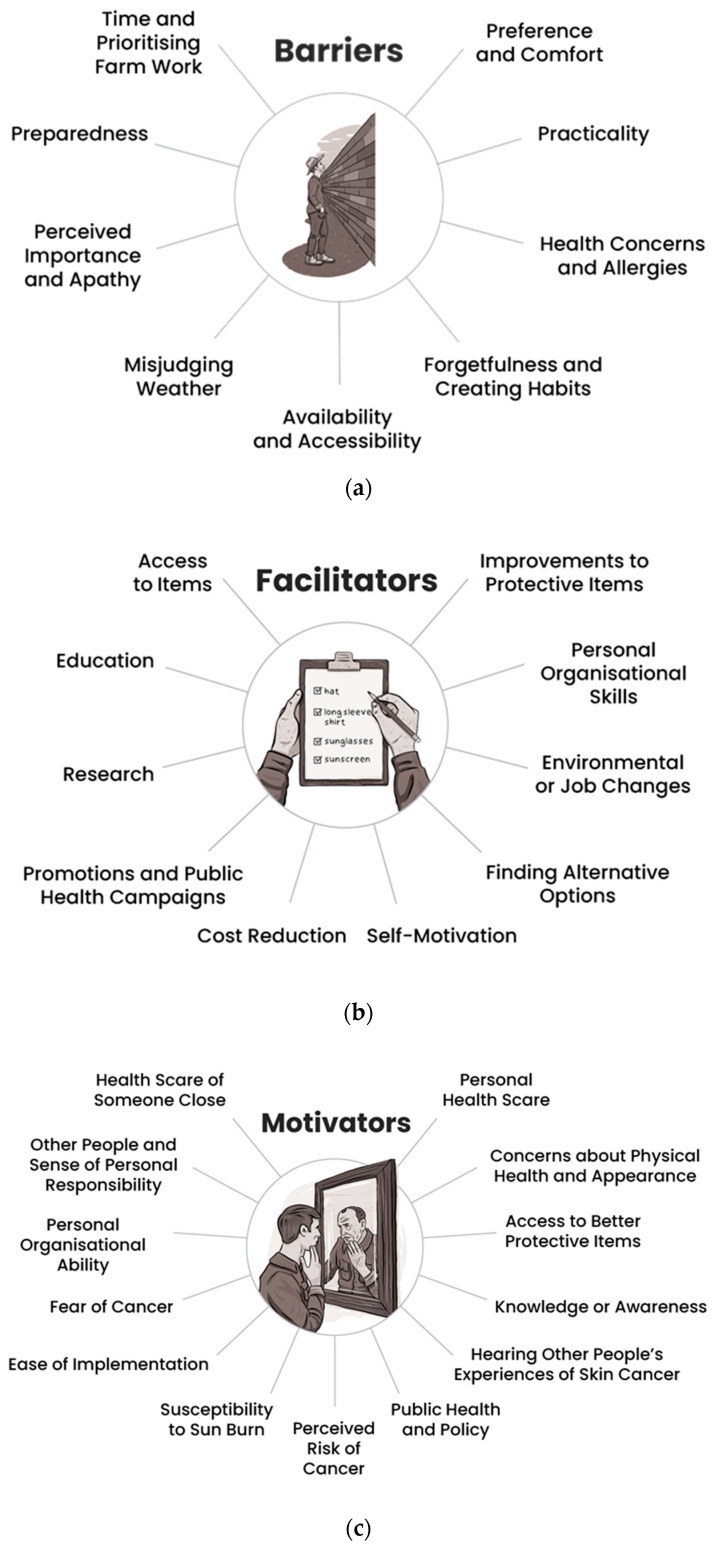
Key barriers (**a**), facilitators (**b**), and motivators (**c**) for performing skin cancer prevention behaviours from the perspective of Australian farmers.

Barriers, facilitators, and motivating influences on prevention behaviours are summarised pictorially in Figure 1. Representative quotes are presented in Table 5. 

Barriers were organised within nine categories: preference and comfort (*n* = 175), practicality (*n* = 169), availability and accessibility (*n* = 116), time and prioritising farm work (*n* = 43), forgetfulness and creating habits (*n* = 36), preparedness (*n* = 34), perceived importance and apathy (*n* = 23), health concerns and allergies (*n* = 21), and misjudging weather (*n* = 6). Many of the barriers described by farmers were specific to the work environment. For example, farmers highlighted that working in the shade was often not an option on the farm. As one farmer stated, “[it’s] hard to be in shade when your work isn’t” (participant 351, male, 40–49 years). Farmers also reported a preference for wearing a peaked cap rather than a wide-brimmed hat because the latter would blow off in the wind or while riding motorbikes. Other farmers noted that wide-brimmed hats restricted their vision, making wearing them potentially hazardous. Long pants and sleeves were reported to be difficult and uncomfortable to work in because they restrict movement and were too hot to wear in the summer heat. Practicality was a major barrier to farmers’ use of sunscreen; they reported that it was “sticky” and caused dust and dirt to stick to the skin. Other barriers related to prioritising farm work over sun safety, forgetting to put sunscreen on or to take it with them to reapply, being prepared for unexpected and changing tasks on the farm, and misjudging the weather or UV intensity.

Facilitators of skin cancer prevention behaviours were organised within 10 categories: improvements to protective items (*n* = 139), personal organisational skills (*n* = 31), access to items (*n* = 30), environmental or job changes (*n* = 20), finding alternative options (*n* = 19), cost reduction (*n* = 11), education (*n* = 9), promotion and public health campaigns (*n* = 7), research (*n* = 4), and self-motivation (*n* = 4). Overwhelmingly, farmers reported that improvements to protective items would facilitate their engagement with skin cancer prevention recommendations. These included clothing that was lightweight and breathable, sunscreen that was not sticky or oily, and hats that were more practical for farm work. Personal organisational skills were another important facilitator, and farmers acknowledged the need to incorporate skin cancer prevention behaviours into their routine. One farmer said that *“it’s actually not that hard—just a matter of getting into the habit of performing said practices” (participant 354, male, 40–49 years)*. One suggestion to help build skin cancer prevention into a farmer’s daily routine was to have a checklist or reminder by the door, on the ute (note: “ute” is an Australian abbreviation for utility vehicle), or in their diary. Several farmers suggested that seeking shade could be facilitated by completing outdoor tasks early in the morning or late in the afternoon to avoid the hottest part of the day. Having sun-protective items easily accessible was another commonly reported facilitator; for example, having sun-protective items stored at multiple worksites, in machinery, and in their ute. Farmers also suggested changes to machinery or yards to reduce sun exposure, including covering sheep and cattle yards, enclosing tractor cabs, planting more trees around the property, or putting shade over workstations.

Finally, factors motivating farmers to make skin cancer prevention a priority were organised into 12 categories: personal health scare (*n* = 113), health scare of someone close (*n* = 54), access to better protective items (*n* = 25), concerns about physical health and appearance (*n* = 22), other people and sense of personal responsibility (*n* = 20), knowledge or awareness (*n* = 17), public health and policy (*n* = 16), susceptibility to sun burn (*n* = 12), ease of implementation (*n* = 8), personal organisational ability (*n* = 8), hearing other people’s experiences of skin cancer (*n* = 7), fear of cancer (*n* = 6), and perceived risk of cancer (*n* = 6). Farmers reported their personal health, including experiencing a cancer scare, to be the primary motivator for engaging with skin cancer prevention. One farmer said that *“getting a touch of it would sharpen my behaviour up” (participant 103, male, 40–49 years)*. Farmers also acknowledged that their age and family history increased their risk of skin cancer. Several farmers said that the appearance of their skin was also motivating. For example, one farmer said that they had noticed their skin looked “more weather beaten” compared to people they knew who worked in offices. Farmers reported that having someone close to them experience a health scare was another potential cue to action. One farmer noted, *“I have not had any close friends or relatives with a bad melanoma experience. Perhaps if I did, I would be more careful” (participant 108, male, 50–59 years).*

## 4. Discussion

Consistent with previous research [22,23,24], our findings suggest that Australian farmers are not adequately engaging with skin cancer prevention behaviours. Sunscreen use was the least frequently ‘always’ employed sun protection strategy; only 6.4% of farmers reported ‘always’ wearing sunscreen during the past summer, and 51.4% reported wearing sunscreen less than half of the time. Although a larger proportion of farmers reported ‘always’ wearing protective sunglasses (44.9%), a wide-brimmed hat (37.7%), and protective clothing (26.3%), sun-protective practices were substantially lower in this South Australian sample of farmers, than reported by farmers in New South Wales in previous research [22]. Seeking shade was more challenging than other sun-protective behaviours, regardless of demographic characteristics, indicative of the practical and environmental barriers to engaging with this strategy for skin cancer prevention in the farming context. Farmers reported a range of barriers to sun protection related to preference and comfort (long pants and sleeves were described as uncomfortable to work in during the summer), practicality (sunscreen is not practical to wear in dusty environments and hats blow off in the wind or restrict vision), accessibility (working in the shade is often not feasible), and time (farm work is prioritised).

Sunscreen use has been a key focus of health promotion campaigns such as SunSmart and is an important strategy for high-risk populations [41], particularly when other methods of sun protection are not available. Importantly, to be effective, sunscreen needs to be regularly reapplied throughout the day. Cancer Council Australia recommend that sunscreen is used on days when the UV Index is forecast to be 3 or above [42]. For context, in Australia, peak daily values regularly exceed 12–14 in summer, and average daily maximum solar UVR levels reach 3 or above in ten months of the year for most of the country [43]. In comparison to other outdoor workers, farmers have been found to have the lowest levels of engagement with sunscreen use [29,31,44,45,46]. Our findings confirmed gender differences described in other studies [24,28,47]; female farmers reported using sunscreen more frequently than males. Interestingly, farmers in our sample who had completed TAFE or trade school were found to use sunscreen more often than those whose highest level of education was high school or university, perhaps indicative of there being a focus on occupational health and safety at TAFE and trade school. Findings also showed that sunscreen use was higher among farmers with a skin type more susceptible to sunburn, as well as those with a personal history of skin cancer.

Smit-Kroner and Brumby [11] suggest that promoting sunscreen is unlikely to result in increased and adequate use among farmers. Consistent with this, farmers in the present study reported that sunscreen was impractical because it is “sticky” and attracts dust and dirt. Fast-drying, non-stick sunscreens targeted towards outdoor workers are becoming more widely available, however there may be a need for more targeted marketing of these products specifically among farmers. Assessment of the suitability of various sunscreens for farm work use may also be beneficial (see Rocholl et al. [48] for methods of assessing sunscreen performance attributes for outdoor work use). Some farmers in the present study also raised health concerns about long-term, daily use of sunscreen. Although research regarding sunscreen safety is continually evolving, there is no clear evidence that long-term use is harmful [49,50], whereas avoidance of sunscreen altogether is likely to have significant negative health implications [51]. Mineral-based sunscreens may be preferred by those with concerns about the safety of chemical-based sunscreens [52].

Cancer Council Australia recommends that sunscreen should be used in combination with other sun protection measures [53]. Farmers may be more responsive to messaging that prioritises strategies that focus on covering the skin (i.e., protective clothing, wide-brimmed hat, protective sunglasses). Smit-Kroner and Brumby [11] argue that protective clothing should be the focus of health promotion messaging targeted to farming populations. Makin, Dobbinson, and Doyle [23] agree that this is a more appropriate form of sun protection for farmers, but state that this must be “balanced by acceptability, usability and safety issues”. How to do this effectively remains an important question; health promotion campaigns that focus on awareness and printed educational resources are often not enough to motivate sustained behaviour change [54,55]. Research among farmers and other outdoor workers demonstrates that knowledge about sun safety does not directly translate into sun-protective behaviour [22,56].

Involving farmers in the design and development of health promotion interventions may increase their efficacy by ensuring that they are culturally appropriate, acceptable, and engaging. Despite being reluctant to seek health advice [25,26], farmers are more receptive to discussing health and wellbeing-related topics among farming industry groups and networks [11], where their unique needs and way of life are understood. Farmers are also adept problem-solvers; those who participated in the present study suggested the following to facilitate engagement with skin cancer prevention behaviours:

- Choosing the best tools for the job—This included wearing clothing that is lightweight and breathable; sunscreen that is not sticky or oily; and hats that are more practical for farm work.
- Getting organised and setting up routines—Organisation could be facilitated by cues to action (sticker prompts, checklists, or reminders) placed strategically by the door, on the ute, or in their diary.
- Completing outdoor tasks early in the morning or late in the afternoon—Reorganisation of daily activities to avoid peak solar UVR exposure.
- Making sun-protective items easily accessible throughout the workday—Ease of use could be improved by having sun-protective items (e.g., sunscreen, wide-brimmed hat, and sunglasses) stored at multiple worksites.
- Adapting the worksite to reduce sun exposure—Simple shade structures, including potentially mobile covers, could be erected and utilised in the most common work areas (e.g., sheep and cattle yards, tractor cabs, and over workstations).

Although individual behaviour change is an essential part of preventing skin cancer, education campaigns targeted to the individual are limited in what they can achieve alone [57,58]. Farmers are likely to benefit from a variety of approaches to support them to implement sun protection into their lives [21]. For example, mandates for sun protection protocols for outdoor workers, tax-deductions for personal protective equipment, and subsidies to help farmers adapt their worksites to reduce occupational sun exposure are initiatives worth exploring. Farming industry groups and networks may also benefit from being trained and supported to run workshops in their local region on farmers’ occupational risk to skin cancer. Additionally, peer-learning should be utilised to bring farmers together to share information about skin cancer, including personal stories, and tips on overcoming barriers to sun-protective practices. Zink et al. [28] suggest that narrative storytelling, a method utilised in other areas of cancer prevention [59], may be a powerful way to communicate culturally appropriate and relevant skin cancer prevention messages to farmers. Narrative messages should emphasise occupational risk, personal health, and sense of personal responsibility (e.g., to family), alongside stories of other people’s experiences with skin cancer.

This study has a number of key strengths. Despite this population being notoriously difficult to reach, we were able to recruit a large number of farmers to participate, giving weight to both the quantitative and qualitative findings. Moreover, findings provide important insights into the frequency with which farmers perform SunSmart behaviours and the characteristics of farmers whose engagement is lowest, highlighting that this is an issue that still requires attention. The qualitative findings provide a richer understanding of the barriers, facilitators, and motivators of skin cancer prevention behaviours and, importantly, provide suggestions from farmers themselves about the individual actions they can take to protect their skin from solar UVR (i.e., a way forward). These learnings could inform the development of peer-led workshops or campaigns to share information about skin cancer prevention in a format that is accessible to farmers and overcomes the barriers they face to engaging with traditional health services [26,60].

Limitations of this study can and should be addressed in future research. Firstly, the sample was limited to South Australian farmers, and mainly contained farmers working on grain, sheep or cattle farms, or sheep and/or cattle properties. Future research should endeavor to recruit participants from other farm types (including but not limited to horticulture, viticulture, and poultry) to explore their perspectives. Secondly, farmers’ reasons for non-participation were not investigated. This information may be useful to inform study design and participant recruitment in future research. Thirdly, this study did not examine the relationship between prior sunburn episodes and performance of SunSmart behaviours. This may be another important variable to include in future research. Finally, it is worth noting that the findings are subject to self-selection and self-reporting biases. It is possible that farmers who responded to the survey were more health conscious and therefore more motivated to engage in sun-protective practices, potentially contributing to an over-estimation of the frequency with which farmers engage in SunSmart behaviours. Findings may also be impacted by recall bias as the survey was conducted during the Australian winter period and respondents were asked to recall their SunSmart behaviours during the previous summer. Nevertheless, farmers’ suggestions for strategies to motivate and facilitate skin cancer prevention, as described in the present study, are valuable for health promotion intervention design and implementation.

## 5. Conclusions

The present study provides important insights into the skin cancer prevention practices of farmers, and describes the barriers, facilitators and motivating influences on skin cancer prevention. Findings indicate a need for sun-protective equipment (e.g., sunscreen, protective clothing, and wide-brimmed hats) that is better suited to the farm work environment, in combination with targeted, culturally appropriate health education messaging to encourage farmers to engage with skin cancer prevention behaviours. Future research should seek to design, implement and evaluate interventions in collaboration with farming groups and communities to improve skin cancer health outcomes for farmers. Community-led educational (e.g., workshops on farmers’ occupational risk to skin cancer) and/or peer-learning (e.g., farmer-led workshops to share information about skin cancer and tips on overcoming barriers to sun-protective practices) approaches that harness the power of narrative storytelling may be effective strategies for promoting skin cancer prevention among Australian farmers.

## Figures and Tables

**Table 1:** Participant characteristics.

Characteristic	N (%) (Unless Indicated Otherwise)
Age (mean, SD)	56.42 (11.05) years
Age range	22–89 years
Gender	
Male	414 (83.1)
Female	57 (11.4)
Marital status	
Married or living with a partner	442 (88.8)
Separated/divorced/widowed	29 (5.8)
Never married	17 (3.4)
Highest education level	
Primary or high school	242 (48.6)
TAFE or trade school	149 (29.9)
University	100 (20.1)
Farm type	
Grain, sheep or cattle	253 (50.8)
Sheep or cattle	175 (35.1)
Dairy or cattle	5 (1.0)
Horticulture	6 (1.2)
Poultry	1 (0.2)
Viticulture	8 (1.6)
Other	49 (9.8)
South Australian region	
Eyre Peninsula	58 (11.6)
Yorke Peninsula	24 (4.8)
Lower/Mid North	88 (17.7)
Far North	19 (3.8)
Murray Mallee	36 (7.2)
South East	144 (28.9)
Kangaroo Island	13 (2.6)
Adelaide Hills or Fleurieu Peninsula	109 (21.9)
Personal history of skin cancer	
Yes—melanoma	27 (5.4)
Yes—non-melanoma	118 (23.7)
No	347 (69.7)
I don’t know	5 (1.0)
Family history of skin cancer	
Yes—melanoma	61 (12.2)
Yes—non-melanoma	170 (34.1)
No	213 (42.8)
I don’t know	40 (8.0)
Fitzpatrick skin photo-type	
Type I—always burn	35 (7.0)
Type II—usually burn	132 (26.5)
Type III—sometimes mild burn	184 (36.9)
Type IV—rarely burn	122 (24.5)
Type V—almost never burn, tan deeply	19 (3.8)
Type VI—almost never burn, deeply pigmented	2 (0.4)

**Table 2:** Frequency of farmers’ skin cancer prevention behaviours performed during the preceding summer.

Behaviour	*N*	1 Never	2	3	4	5	6	7 Always
Wearing protective clothing	490	8.6%	8.2%	9.4%	12.0%	16.7%	18.8%	26.3%
Wearing SPF 30+ (or higher) sunscreen	483	16.6%	20.9%	13.9%	15.5%	12.8%	13.9%	6.4%
Wearing a wide-brimmed hat	491	5.9%	7.3%	7.3%	8.4%	13.4%	20.0%	37.7%
Seeking shade	480	2.7%	7.5%	9.0%	23.8%	25.8%	18.1%	13.1%
Wearing protective sunglasses	486	10.5%	5.3%	4.9%	9.3%	9.7%	15.4%	44.9%

**Table 3:** Gender differences in skin cancer prevention behaviours (Mann–Whitney *U* test).

	Gender				
	Male (Mean Rank)	Female (Mean Rank)	Mann–Whitney *U*	Z-Value	*p*-Value
Wearing protective clothing	231.68	238.34	11,266.5	−0.357	0.721
Wearing SPF 30+ (or higher) sunscreen	217.58	313.38	6647.5	−5.180	0.000
Wearing a wide-brimmed hat	234.92	215.50	10,613.5	−1.075	0.282
Seeking shade	224.14	255.53	9630.5	−1.707	0.088
Wearing protective sunglasses	228.04	247.89	10,494	−1.112	0.266

**Table 4:** Differences in skin cancer prevention behaviours between groups based on age, education, personal and family history of skin cancer, farm type, and Fitzpatrick skin photo-type category (Kruskal–Wallis H test).

	Age		Education		Personal History		Family History		Farm Type		Fitzpatrick Skin Phototype	
	χ^2^	*p*-Value	χ^2^	*p*-Value	χ^2^	*p*-Value	χ^2^	*p*-Value	χ^2^	*p*-Value	χ^2^	*p*-Value
Wearing protective clothing	18.430	0.005	2.114	0.347	5.166	0.076	4.874	0.181	5.069	0.408	21.663	0.001
Wearing SPF 30+ (or higher) sunscreen	11.135	0.084	7.772	0.021	9.424	0.009	6.975	0.073	1.484	0.915	39.676	0.000
Wearing a wide-brimmed hat	13.605	0.034	0.601	0.740	18.869	0.000	7.179	0.066	4.078	0.538	9.686	0.085
Seeking shade	10.776	0.096	1.006	0.605	1.922	0.383	0.636	0.888	10.374	0.065	3.393	0.640
Wearing protective sunglasses	21.046	0.002	1.882	0.390	1.610	0.447	0.458	0.928	3.462	0.629	9.366	0.095

**Table 5:** Barriers, facilitators, and motivating influences for skin cancer prevention behaviours as reported by Australian farmers.

Categories	n	%	Representative Quotes
Barriers ^1^			
Preference and comfort	175	28.1	*“It is often too hot in summer to wear long pants or a shirt with long sleeves” (participant 160, male, 70–79 years)*
Practicality	169	27.1	*“Wide-brimmed hats are useless on motorbikes. Better to wear a cap that you can wear 100% of the time.” (participant 237, male, 40–49 years)*
Availability and accessibility	116	18.6	*“Not always possible to work in the shade depending on task,* e.g., *moving cattle, feeding cattle” (participant 224, female, 60–65 years)*
Time and prioritising farm work	43	6.9	*“Farmers are constantly on the run at harvest time doing a lot of different jobs, so occasionally [skin cancer] prevention gets delayed” (participant 26, male, 50–59 years)*
Forgetfulness and creating habits	36	5.8	*“I tend to forget to put on sunscreen, and if I remember it’s usually when I feel my skin getting hot” (participant 417, female, 30–39 years)*
Preparedness	34	5.5	*“Not organised enough to have sunscreen available other than in farmhouse where it keeps better” (participant 268, male, 60–69 years*
Perceived importance and apathy	23	3.7	*“…can’t be bothered taking the time to seek protection” (participant 329, male, 50–59 years)*
Health concerns and allergies	21	3.4	*“…daily application of sunscreen in the long term could cause health issues in itself” (participant 321, male, 60–69 years)*
Misjudging weather	6	0.9	*“Sometimes I misjudge the intensity of the sun, even on cooler days” (participant 459, male, 50–59 years)*
Facilitators ^2^			
Improvements to protective items	139	50.7	*“Suitably designed sun-protective work [clothes] that [are] light and comfortable to wear in the warmest of weather.” (participant 37, male, 50–59 years)*
Personal organisational skills	31	11.3	*“Putting reminder stickers on iPads, ute windscreens, diaries, etc.” (participant 435, female, 60–69 years)*
Access to items	30	10.9	*“Have a stockpile of sunscreen in Eski to keep cool in car and purchase many wide-brimmed hats [with] ventilation” (participant 377, male, 50–59 years)*
Environmental or job changes	20	7.3	*“Probably the best way for me to see less sun is to build a cover over the sheep yards” (participant 164, gender not disclosed, 50–59 years)*
Finding alternative options	19	6.9	*“Spray sunscreens make it easier to reapply when your hands and skin are dirty and sweaty” (participant 158, female, 30–39 years)*
Cost reduction	11	4.0	*“Make quality sunglasses more affordable” (participant 58, gender not disclosed, 50–59 years)*
Education	9	3.3	*“Must retrain the dog. Easy to train a puppy not so for the dog.” (participant 389, male, 60–69 years)*
Promotions and public health campaigns	7	2.6	*“Many farmers I see wear caps rather than [wide-brimmed hats]. Most of these caps have brand names and would be promotional. A greater use of [wide-brimmed hats] by companies would help change what people wear.” (participant 430, male, 50–59 years)*
Research	4	1.5	*“It would be easier to use sunscreen if it was known to be absolutely safe to use repeatedly” (participant 218, male, 70–79 years)*
Self-motivation	4	1.5	*“A change of mindset on the discomfort of sweating in long sleeves and pants over the benefits gained” (participant 239, male, 70–79 years)*
Motivating factors ^3^			
Personal health scare	113	35.9	*“Getting a touch of it would sharpen my behaviour up” (participant 103, male, 40–49 years)*
Health scare of someone close	54	17.2	*“I have not had any close friends or relatives with a bad melanoma experience. Perhaps if I did, I would be more careful.” (participant 180, male, 50–59 years)*
Access to better protective items	25	7.9	*“Cooler clothing and more practical hat” (participant 16, male, 60–69 years)*
Concerns about physical health and appearance	22	7.0	*“Making me look a lot older than I am. This was evident to me at a class reunion where I saw classmates after 30 years. The ones with office jobs had a lot better skin than me, and I looked more weather beaten.” (participant 260, male, 50–59 years)*
Other people and sense of personal responsibility	20	6.4	*“A better sense of personal responsibility to the risks and grief it would cause myself and my family” (participant 355, male, 60–69 years)*
Knowledge or awareness	17	5.4	*“Knowledge about the reality of skin cancer and its devastating effects” (participant 382, female, 60–69 years)*
Public health and policy	16	5.1	*“I have seen self-help check sheets. [Distribute at] silos, hotels, health centres, CFS, SES, IT centres, schools, merchandise agents (Elders), Field Days, Post [Office]…” (participant 206, male, 70–79 years)*
Susceptibility to sun burn	12	3.8	*“Knowing my skin type puts me at greater risk of sun damage and skin cancer” (participant 60, male, 60–69 years)*
Ease of implementation	8	2.6	*“If it was easy and worked with my work practices” (participant 325, male, 30–39 years)*
Personal organisational ability	8	2.6	*“Plan[ning] ahead” (participant 18, male, 50–59 years)*
Hearing other people’s experiences of skin cancer	7	2.2	*“Hearing from and seeing people who have gone through the trauma of skin cancer” (participant 249, male, 40–49 years)*
Fear of cancer	6	1.9	*“Fear of skin cancer and its consequences motivate me” (participant 37, male, 50–59 years)*
Perceived risk of cancer	6	1.9	*“My own perceived risk to an occurrence [of skin cancer]” (participant 379, male, 60–69 years)*

^1^ Responses to open-ended question: “What made performing one or more of the skin cancer prevention behaviours outlined above difficult for you last summer?”. ^2^ Responses to open-ended question: “How could the skin cancer prevention behaviours listed above be made easier to perform?”. ^3^ Responses to open-ended question: “What would motivate you to make preventing skin cancer a priority?”.

## Data Availability

The data presented in this study are available on request from the corresponding author.

## Supplementary Materials

The following supporting information can be downloaded at: https://www.mdpi.com/article/10.3390/ijerph19052940/s1, Figure S1: Coding tree for barriers to prevention; Figure S2: Coding tree for facilitators of prevention; Figure S3: Coding tree for motivating factors for prevention; Table S1: Barriers to skin cancer prevention behaviours as reported by farmers; Table S2: Facilitators of skin cancer prevention behaviours as reported by farmers; Table S3: Motivating factors for skin cancer prevention behaviours as reported by farmers.
