# Supplementary material for: “She’ll Be Right, Mate”: A Mixed Methods Analysis of Skin Cancer Prevention Practices among Australian Farmers—An At-Risk Group
Source: Int J Environ Res Public Health. 2022 Mar 3;19(5):2940. doi: 10.3390/ijerph19052940 (PMC8910209; doi:10.3390/ijerph19052940)
Supplement: Supplementary file 1 [file ijerph-19-02940-s001.zip › ijerph-1607664-supplementary.pdf]

“She’ll be right, mate”: A mixed methods analysis of skin cancer prevention practices among Australian farmers – an at-risk group

Supplementary Materials

Figure S1. Coding tree for barriers to prevention.

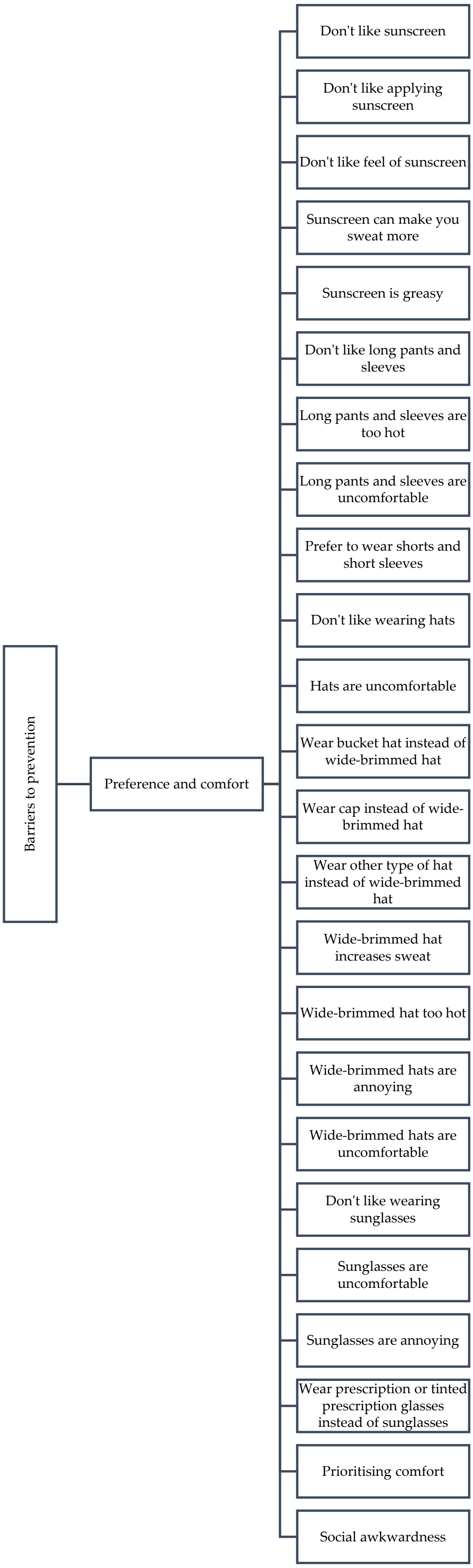

Figure S1. continued.

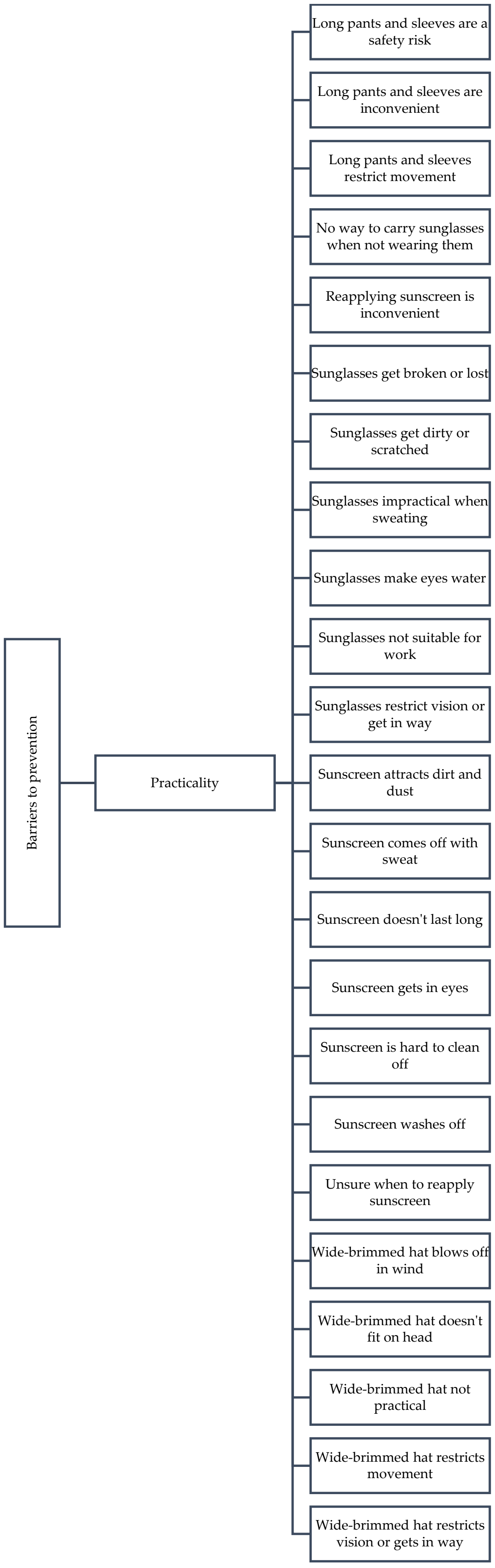

Figure S1. continued.

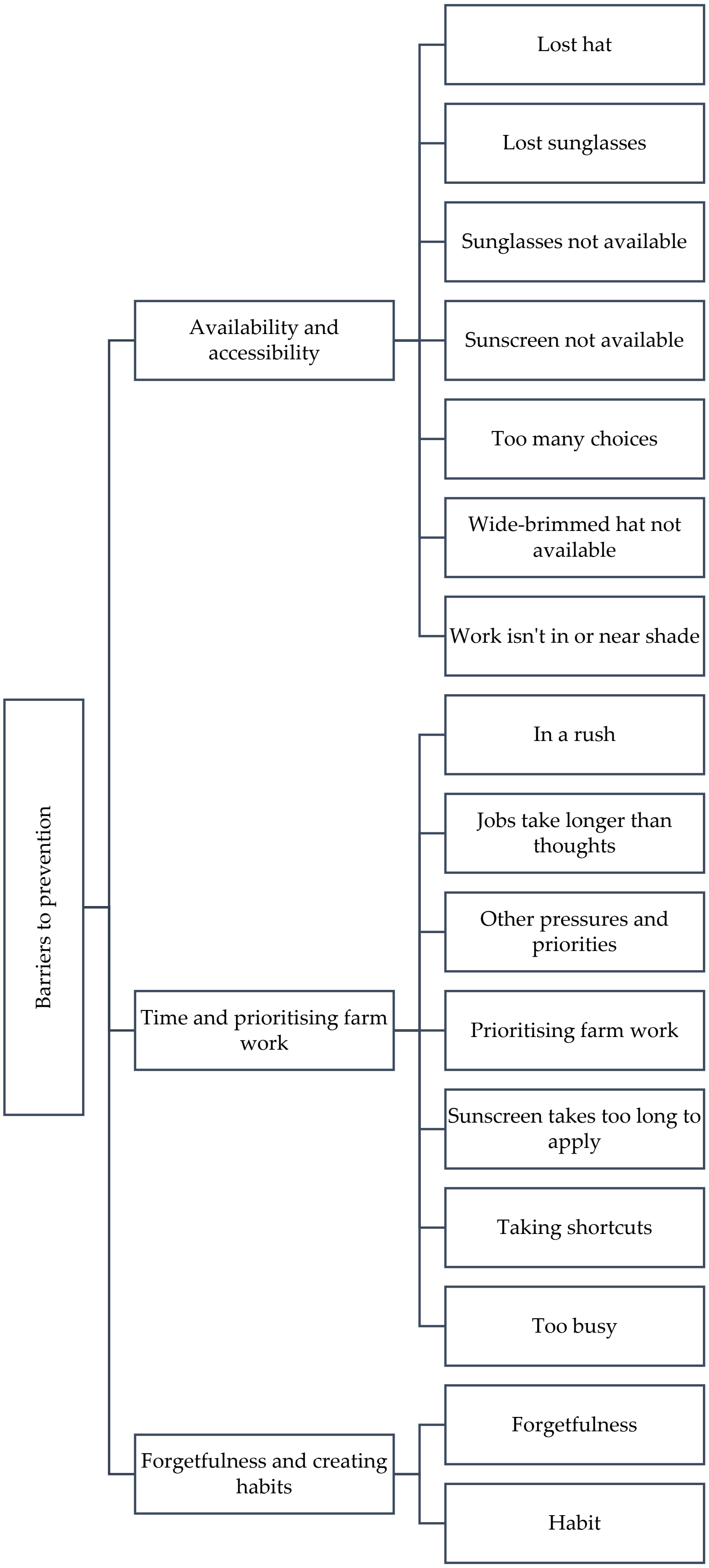

Figure S1. continued.

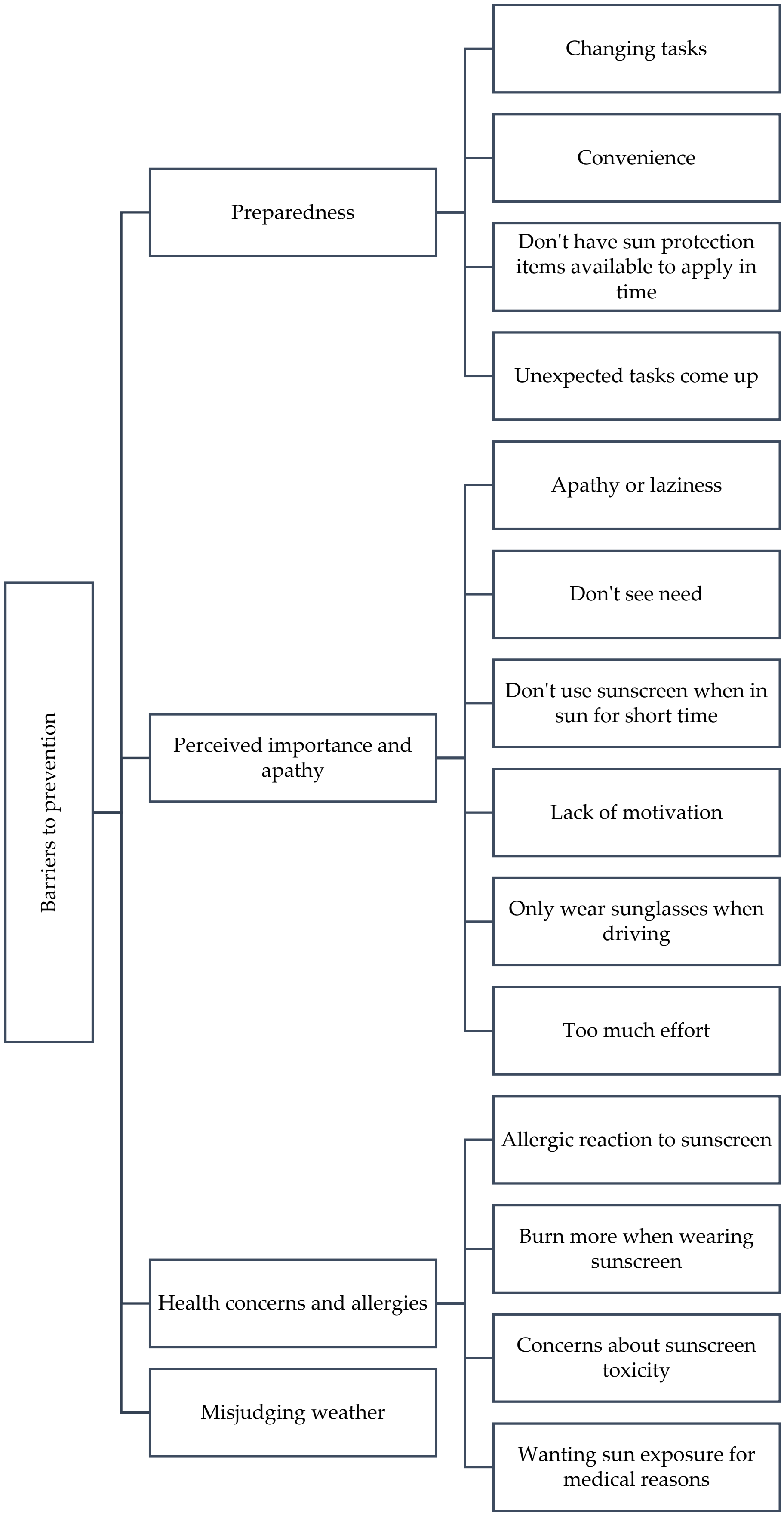

Figure S2. Coding tree for facilitators of prevention.

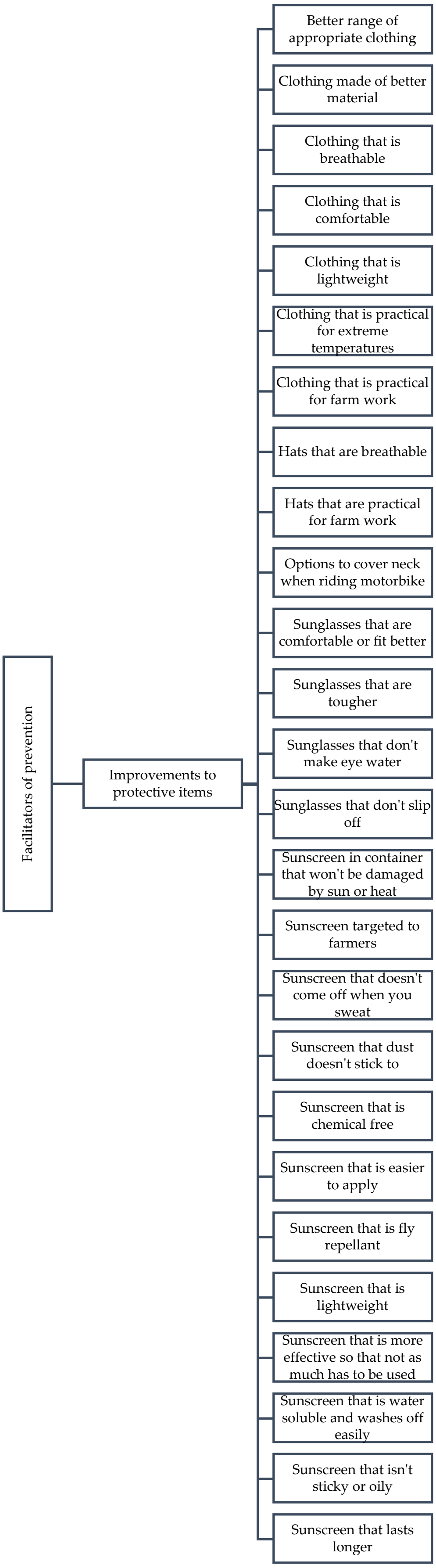

Figure S2. continued.

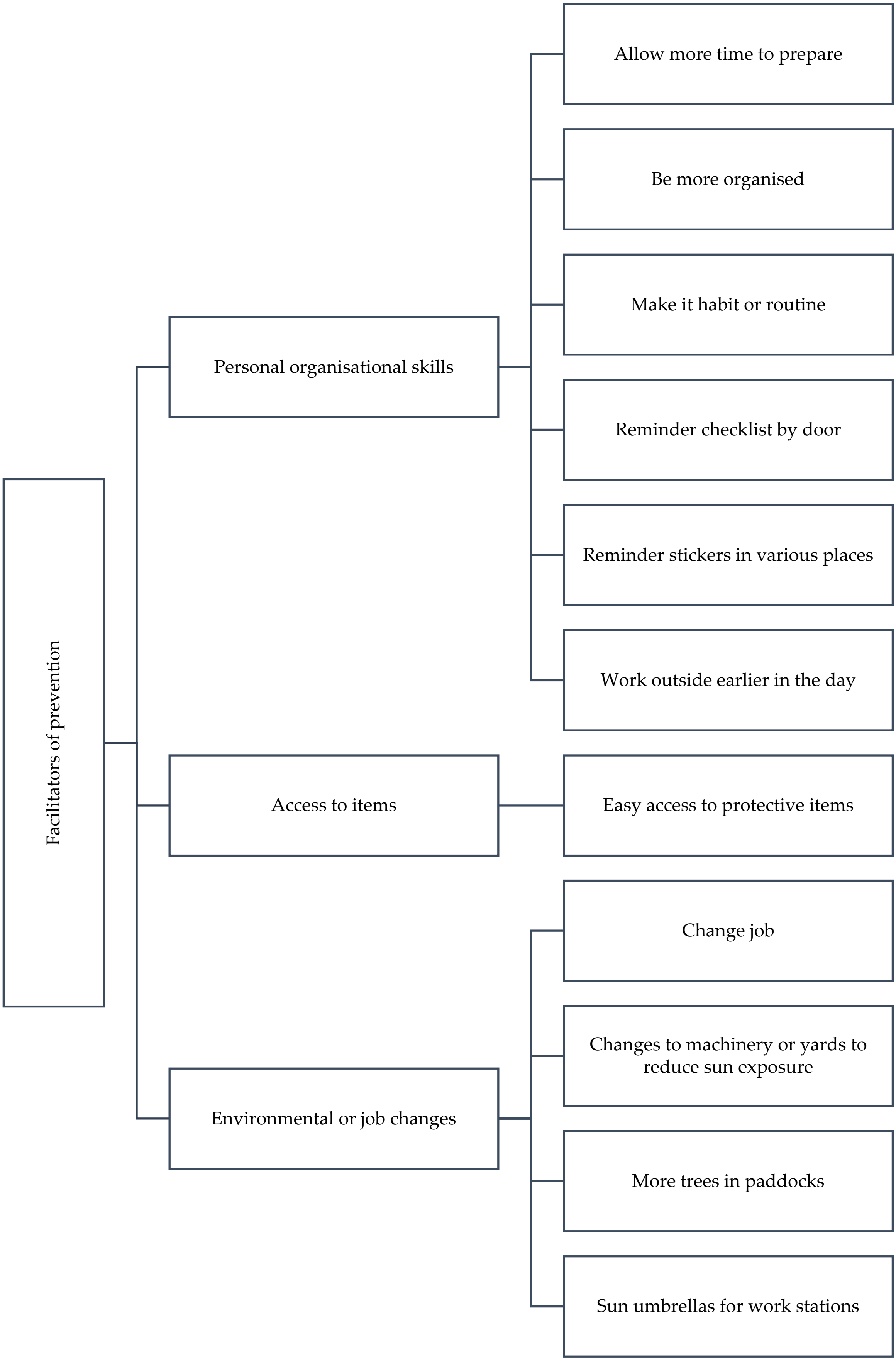

Figure S2. continued.

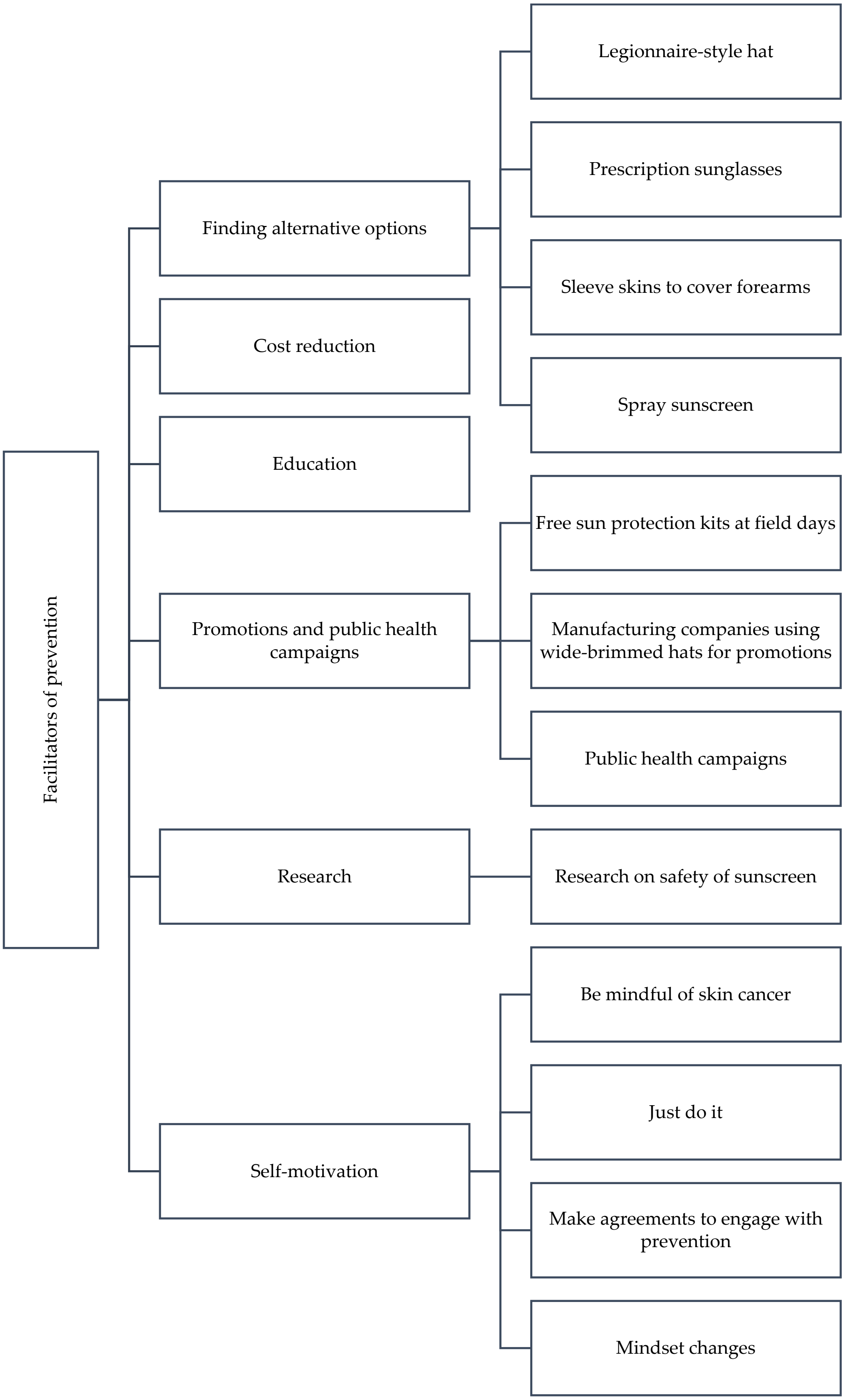

Figure S3. Coding tree for motivating factors for prevention

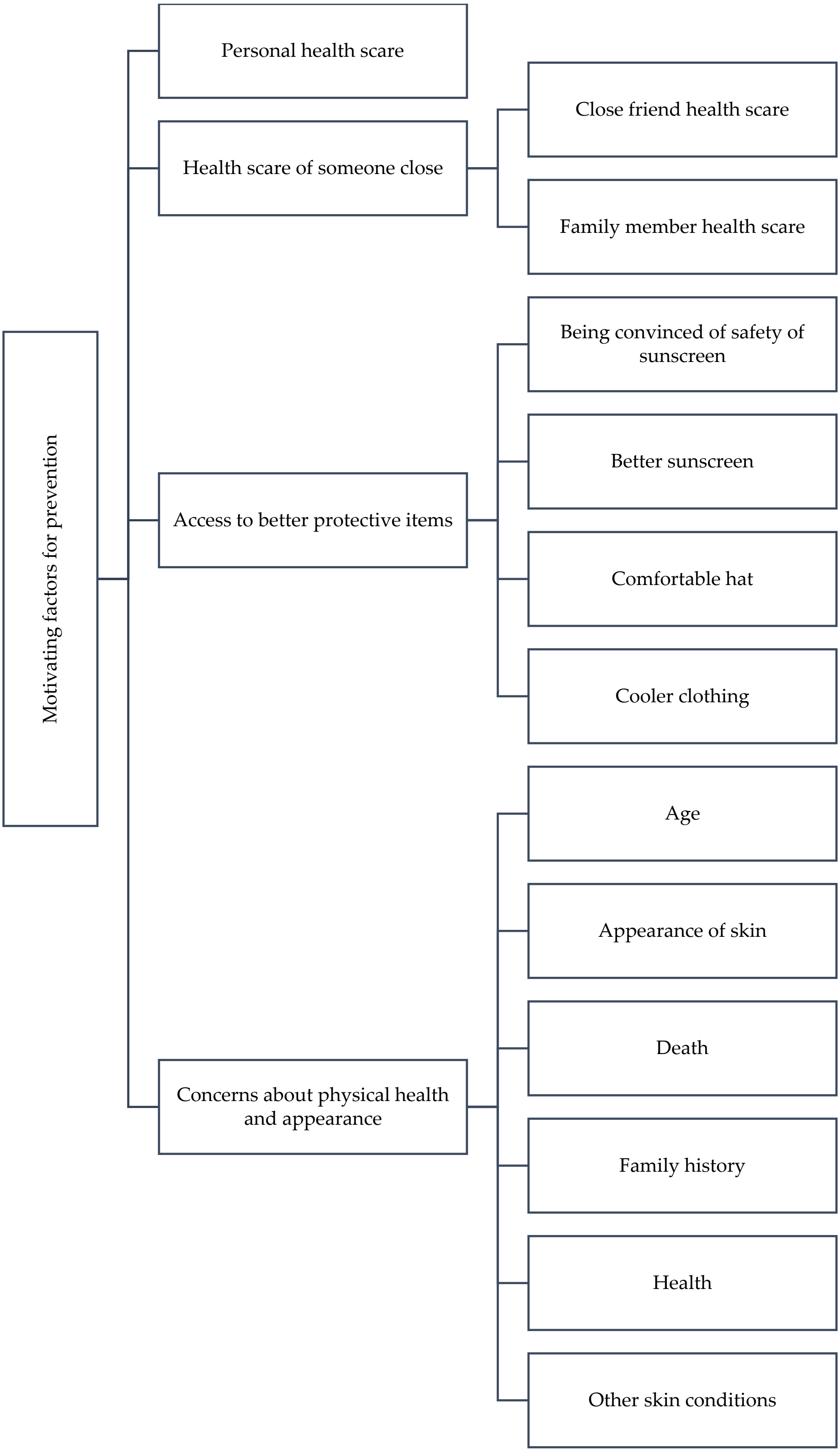

Figure S3. continued.

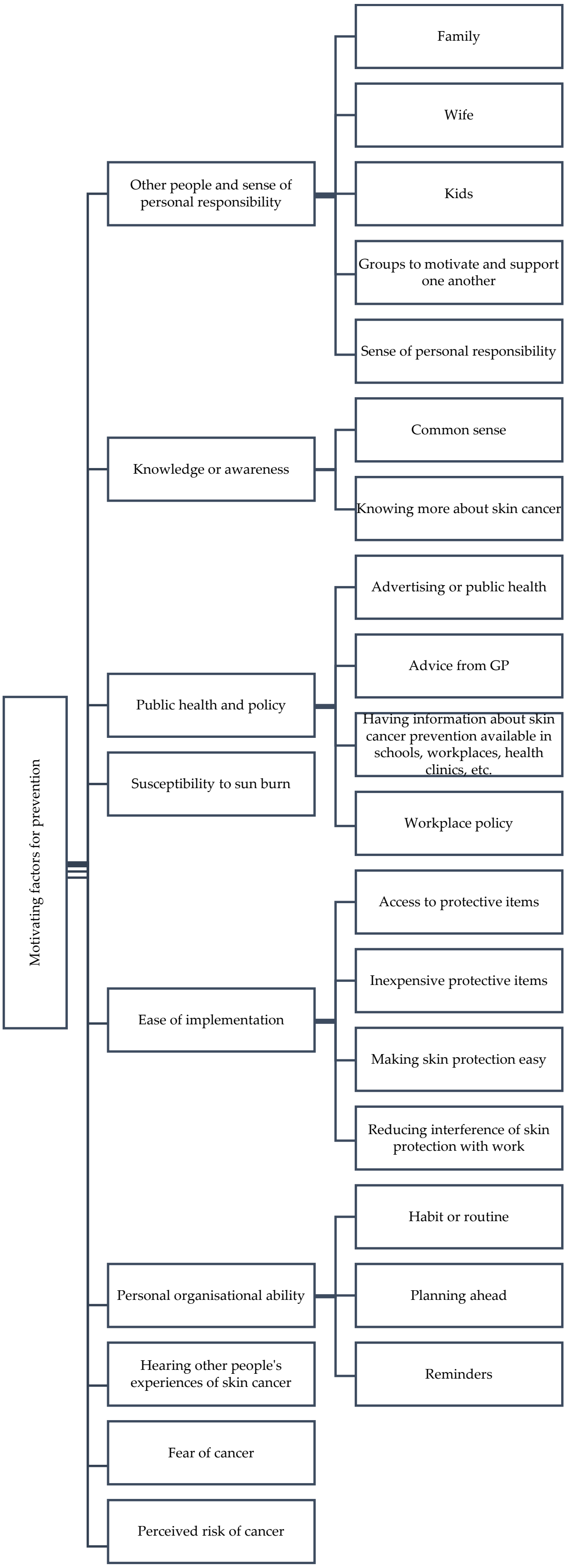

Table S1. Barriers to skin cancer prevention behaviours as reported by farmers.[1]

Type of barrier	n	%	Definition and representative quotes
Preference and comfort	175	28.1	Barriers relating to personal preferences and comfort of sun-protective items.<br><br>*"It is often too hot in summer to wear long pants or a shirt with long sleeves" (participant 160, male, 70-79 years)*<br><br>*"Do not wear long pants – intolerable in heat" (participant 62, male, 60-69 years)*<br><br>*"Dislike greasy sunscreen, which to me makes natural perspiration more difficult" (participant 122, male, 50-59 years)*<br><br>*"I wear mostly a form of peak[ed] cap because wide-brim[med] hats make me sweat" (participant 187, male, 50-59 years)*<br><br>*"Sunglasses don't fit well over my normal glasses" (participant 403, female, 50-59 years)*
Practicality	169	27.1	Barriers relating to the practicality of sun-protective items for work on the farm.<br><br>*"Long sleeve shirts are difficult to work in, more dangerous with machinery and hotter to wear" (participant 11, male, age not disclosed)*<br><br>*"Wide-brimmed hats are useless on motorbikes. Better to wear a cap that you can wear 100% of the time." (participant 237, male, 40-49 years)*<br><br>*"Dust and dirt sticks to sunscreen and makes it uncomfortable" (participant 208, male, 50-59 years)*<br><br>*"I find sunscreen sticky and during harvest dust sticks to your face and arms" (participant 483, male, 50-59 years)*<br><br>*"Big floppy hats reduce visibility when feeding out hay in large groups of horses. Need to watch out for horses kicking at other horses, near me. So I wear a peaked cap which I realise is not as protective." (participant 171, female, 60-69 years)*
Availability and accessibility	116	18.6	Barriers relating to availability and accessibility of sun-protective items.<br><br>*"Don't always carry sunscreen, particularly when working on motorbike" (participant 71, male, 50-59 years)*

Type of barrier	n	%	Definition and representative quotes
			"Not always possible to work in the shade depending on task, e.g. moving cattle, feeding cattle" (participant 224, female, 60-69 years)<br><br>*"Always being outside on the property – too far from house to reapply [sunscreen] or cover up" (participant 72, female, 50-59 years)*<br><br>*"Hard to be in shade when your work isn't" (participant 351, male, 40-49 years)*
Time and prioritising farm work	43	6.9	Barriers relating to busyness and prioritizing farm work.<br><br>*"Farmers are constantly on the run at harvest time doing a lot of different jobs, so occasionally [skin cancer] prevention gets delayed" (participant 26, male, 50-59 years)*<br><br>*"Always in a hurry" (participant 64, male, 40-49 years)*
Forgetfulness and creating habits	36	5.8	Barriers relating to forgetfulness or the need to create habits around use of sun-protective items.<br><br>*"I tend to forget to put on sunscreen, and if I remember it's usually when I feel my skin getting hot" (participant 417, female, 30-39 years)*<br><br>*"Forgot to take sunscreen to reapply" (participant 150, female, 60-69 years)*<br><br>*"Childhood habits – SunSmart didn't exist when I was a child. Trying to develop habit, but still need prodding" (participant 389, male, 60-69 years)*
Preparedness	34	5.5	Barriers relating to being organized or prepared for changing tasks on the farm.<br><br>*"Not organised enough to have sunscreen available other than in farmhouse where it keeps better" (participant 268, male, 60-69 years)*<br><br>*"Some unexpected stock problem arising which had to be fixed and no protection at hand" (participant 373, male, 70-79 years)*
Perceived importance and apathy	23	3.7	Barriers relating to the perceived importance of, and apathy to, skin cancer prevention behaviours.<br><br>*"…can't be bothered taking the time to seek protection" (participant 459, male, 50-59 years)*<br><br>*"…just lazy and need to get more organised" (participant 318, female, 50-59 years)*

Type of barrier	n	%	Definition and representative quotes
			"Old adage – she'll be right, mate" (participant 44, male, 60-69 years)
			"…just don't see the need" (participant 323, female, 70-79 years)
			"I don't burn so I don't use any protection" (participant 101, male, 30-39 years)
			"Applying sunscreen constantly was too much effort" (participant 481, male, 30-39 years)
Health concerns and allergies	21	3.4	Barriers relating to health concerns or allergies to sunscreen.
			"Skin very sensitive to sunscreen" (participant 81, male, 50-59 years)
			"…daily application of sunscreen in the long term could cause health issues in itself" (participant 321, male, 60-69 years)
			"…worry a little about the chemicals in sunscreen…" (participant 134, female, 40-49 years)
Misjudging weather	6	0.9	Barriers relating to misjudging the weather or UV intensity.
			"Sometimes I misjudge the intensity of the sun, even on cooler days" (participant 459, male, 50-59 years)
			"Changes in cloud cover / sun intensity" (participant 130, male, 60-69 years)

[1] Responses to open-ended question: "What made performing one or more of the skin cancer prevention behaviours outlined above difficult for you last summer?"

Table S2. Facilitators of skin cancer prevention behaviours as reported by farmers.[2]

Type of facilitator	n	%	Definition and representative quotes
Improvements to protective items	139	50.7	Facilitators relating to making improvements to sun-protective items to be better-suited to farm work.<br><br>*"Suitably designed sun protective work [clothes] that [are] light and comfortable to wear in the warmest of weather." (participant 37, male, 50-59 years)*<br><br>*"Make sunscreen easer to apply and less oily, with a build-in insect repellant." (participant 264, gender not disclosed, 40-49 years)*<br><br>*"Cooler and less uncomfortable protective clothing" (participant 228, male, 60-69 years)*
Personal organisational skills	31	11.3	Facilitators relating to being better organised.<br><br>*"It's actually not that hard, just a matter of getting into the habit of performing said practices." (participant 354, male, 40-49 years)*<br><br>*"Work earlier and later in the day. Try to avoid noon 'til 4PM." (participant 200, male, 60-69 years)*<br><br>*"Putting reminder stickers on iPads, ute windscreens, diaries, etc." (participant 435, female, 60-69 years)*
Access to items	30	10.9	Facilitators relating to having access to sun-protective items.<br><br>*"Have a stockpile of sunscreen in Eski to keep cool in car and purchase many wide-brimmed hats [with] ventilation" (participant 377, male, 50-59 years)*<br><br>*"Carry sunscreen in my lunchbox and apply to my hands and face during each break – make this a 'habit' on high UV days!" (participant 272, male, 50-59 years)*<br><br>*"Have sunscreen, hat, and glasses at back door as you leave home" (participant 192, male, 60-69 years)*
Environmental or job changes	20	7.3	Facilitators relating to making changes to the work environment.<br><br>*"Probably the best way for me to see less sun is to build a cover over the sheep yards" (participant 164, gender not disclosed, 50-59 years)*<br><br>*"By planting more trees … to provide more share for myself and the cattle" (participant 160, male, 70-79 years)*

Type of facilitator	n	%	Definition and representative quotes
			"Sturdy sun umbrellas for workstations" (participant 369, male, 60-69 years)
Finding alternative options	19	6.9	Facilitators relating to finding alternative sun-protective items that are better-suited to farm work.<br><br>*"Spray sunscreens make it easier to reapply when your hands and skin are dirty and sweaty" (participant 158, female, 30-39 years)*<br><br>*"Prescription sunglasses" (participant 228, male, 60-69 years)*<br><br>*"Sleeve-like skins to cover forearms rather than loose sleeves" (participant 221, male, 30-39 years)*
Cost reduction	11	4.0	Facilitators relating to reducing the cost of sun-protective items.<br><br>*"Access to a range of clothing suited to hot, dusty work that is easy to wear and reasonably fashionable, without spending a fortune." (participant 388, male, 50-59 years)*<br><br>*"Make quality sunglasses more affordable" (participant 54, gender not disclosed, 50-59 years)*
Education	9	3.3	Facilitators relating to educating farmers about skin cancer prevention.<br><br>*"Must retrain the dog. Easy to train a puppy not so for the dog." (participant 389, male, 60-69 years)*<br><br>*"Educate the young – didn't happen when I was young – big sunburn every summer." (participant 440, male, 80-89 years)*
Promotions and public health campaigns	7	2.6	Facilitators relating to promoting skin cancer prevention and running public health campaigns.<br><br>*"Industry manufacturers supplies often provide free caps. I have never seen any free wide-brimmed hats, which could still have company logo." (participant 162, male, 60-69 years)*<br><br>*"Many farmers I see wear caps rather than [wide-brimmed hats]. Most of these caps have brand names and would be promotional. A greater use of [wide-brimmed hats] by companies would help change what people wear." (participant 430, male, 50-59 years)*<br><br>*"…maybe more surveys or [skin cancer] prevention kits could be given out at Field Days or local shows." (participant 187, male, 50-59 years)*<br><br>*"Advertising programs" (participant 490, male, 70-79 years)*

Type of facilitator	n	%	Definition and representative quotes
			"Advising public of skin cancer statistics, especially fatalities" (participant 490, male, 70-79 years)
Research	4	1.5	Facilitators relating to research into the safety of sunscreen.
			"Determine and publish risks associated with wearing sunscreen" (participant 349, male 70-79 years)
			"It would be easier to use sunscreen if it was known to be absolutely safe to use repeatedly" (participant 218, male, 70-79 years)
Self-motivation	4	1.5	Facilitators relating to self-motivation.
			"Firmly resolve to take all listed behaviours" (participant 65, male, 40-49 years)
			"A change of mindset on the discomfort of sweating in long sleeves and pants over the benefits gained" (participant 239, male, 70-79 years)

[2] Responses to open-ended question: "How could the skin cancer prevention behaviours listed above be made easier to perform?"

Table S3. Motivating factors for skin cancer prevention behaviours as reported by farmers.[3]

Type of motivating factor	n	%	Definition and representative quotes
Personal health scare	113	35.9	Motivating factors relating to a personal health scare.<br><br>*"A positive diagnosis would get me thinking that I should know better" (participant 109, male, 50-59 years)*<br><br>*"Getting a touch of it would sharpen my behaviour up" (participant 103, male, 40-49 years)*
Health scare of someone close	54	17.2	Motivating factors relating to a health scare of a family member, friend, or colleague.<br><br>*"I have not had any close friends or relatives with a bad melanoma experience. Perhaps if I did, I would be more careful." (participant 180, male, 50-59 years)*<br><br>*"Probably if someone close to me was diagnosed with skin cancer and was following the same preventative practices that I am (would then have to question to effectiveness of my prevention strategies)." (participant 327, male, 40-49 years)*
Access to better protective items	25	7.9	Motivating factors relating to access to better sun-protective items.<br><br>*"More suitable sunscreen products" (participant 35, male, 60-69 years)*<br><br>*"Cooler clothing and more practical hat" (participant 16, male, 60-69 years)*
Concerns about physical health and appearance	22	7.0	Motivating factors relating to physical health (e.g., age) or appearance (e.g., appearance of sun damage).<br><br>*"I really know I should, I should make it all routine to put on sunscreen every day. I look at my skin and feel my hands and neck are looking aged because of being outdoors all the time, this should be enough, but I guess it's not." (participant 417, female, 30-39 years)*<br><br>*"Making me look a lot older than I am. This was evident to me at a class reunion where I saw classmates after 30 years. The ones with office jobs had a lot better skin than me, and I looked more weather beaten." (participant 260, male, 50-59 years)*
Other people and personal responsibility	20	6.4	Motivating factors relating to other people (family, spouse, kids) and a sense of personal responsibility.<br><br>*"My wife's nagging" (participant 410, male, 50-59 years)*

Type of motivating factor	n	%	Definition and representative quotes
			"A better sense of personal responsibility to the risks and grief it would cause myself and my family" (participant 355, male, 60-69 years)<br><br>*"Being a good example to staff and family and leading by always wearing PPE in summer heat." (participant 139, male, 50-59 years)*
Knowledge or awareness	17	5.4	Motivating factors relating to knowledge or awareness of skin cancer and skin cancer prevention behaviours.<br><br>*"Knowledge about the reality of skin cancer and its devastating effects" (participant 382, female, 60-69 years)*<br><br>*"Understanding the repercussions of not preventing it" (participant 363, male, 60-69 years)*
Public health and policy	16	5.1	Motivating factors relating to public health advice and workplace policy.<br><br>*"…a little flyer through the post every few years would be enough to 'alert' me. In particular, the benefits of sunscreen." (participant 272, male, 50-59 years)*<br><br>*"…seeking the advice of a doctor." (participant 475, male, 80-89 years)*<br><br>*"I have seen self-help check sheets. [Distribute at] silos, hotels, health centres, CFS, SES, IT centres, schools, merchandise agents (Elders), Field Days, Post [Office]…" (participant 206, male, 70-79 years)*<br><br>*"Push that skin cancer is an OHS issue, same as breathing chemicals." (participant 430, male, 50-59 years)*
Susceptibility to sun burn	12	3.8	Motivating factors relating to personal susceptibility to sun burn.<br><br>*"Knowing my skin type puts me at greater risk of sun damage and skin cancer" (participant 60, male, 60-69 years)*<br><br>*"Being susceptible to sun burn" (participant 8, male, 50-59 years)*
Ease of implementation	8	2.6	Motivating factors relating to the ease of implementing skin cancer prevention behaviours.<br><br>*"If it was easy and worked with my work practices" (participant 325, male, 30-39 years)*

Type of motivating factor	n	%	Definition and representative quotes
			"Finding the correct attire easily and not too expensive" (participant 388, male, 50-59 years)<br><br>*"Having the hat and sunscreen available" (participant 303, female, 40-49 years)*<br><br>*"Making it easy" (participant 163, male, 50-59 years)*
Personal organisational ability	8	2.6	Motivating factors relating to being better organised (e.g., creating habits, planning ahead, having reminders, etc.).<br><br>*"Incorporation into daily routine" (participant 425, female, 40-49 years)*<br><br>*"Plan[ning] ahead" (participant 18, male, 50-59 years)*
Hearing other people's experiences of skin cancer	7	2.2	Motivating factors relating to hearing other people's experiences of skin cancer.<br><br>*"Hearing from and seeing people who have gone through the trauma of skin cancer" (participant 249, male, 40-49 years)*<br><br>*"Seeing images and results of cancer" (participant 29, male, 50-59 years)*<br><br>*"Someone my age getting affected by skin cancer due to UV exposure" (participant 448, male, 30-39 years)*
Fear of cancer	6	1.9	Motivating factors relating to fear of cancer.<br><br>*"Fear of skin cancer and its consequences motivate me" (participant 37, male, 50-59 years)*<br><br>*"The fear of getting a serious malignant melanoma" (participant 123, male, 50-59 years)*
Perceived risk of cancer	6	1.9	Motivating factors relating to the perceived risk of skin cancer.<br><br>*"My own perceived risk to an occurrence [of skin cancer]" (participant 379, male, 60-69 years)*<br><br>*"Statistics on workers in our industry" (participant 322, male, 40-49 years)*

[3] Responses to open-ended question: "What would motivate you to make preventing skin cancer a priority?"
